# Commentary on COVID-19 Vaccine Hesitancy in sub-Saharan Africa

**DOI:** 10.3390/tropicalmed7070130

**Published:** 2022-07-11

**Authors:** Severin Kabakama, Eveline T. Konje, Jerome Nyhalah Dinga, Colman Kishamawe, Imran Morhason-Bello, Peter Hayombe, Olufela Adeyemi, Ernest Chimuka, Ivan Lumu, John Amuasi, Theophilus Acheampong, Tafadzwa Dzinamarira

**Affiliations:** 1 Humanitarian and Public Health Consultant, Mwanza P.O. Box 511, Tanzania; 2 Department of Biostatistics, Epidemiology and Behavioral Sciences, School of Public Health, Catholic University of Health and Allied Sciences, Mwanza P.O. Box 1464, Tanzania; ekonje28@bugando.ac.tz; 3 Michael Gahnyam Gbeugvat Foundation, Buea P.O. Box 63, Cameroon; djnyhalah@yahoo.com; 4 Biotechnology Unit, University of Buea, Buea P.O. Box 63, Cameroon; 5 National Institute for Medical Research, Mwanza P.O. Box 1462, Tanzania; kishamawe@yahoo.com; 6 Department of Obstetrics & Gynaecology, Faculty of Clinical Sciences, Institute for Advanced Medical Research and Training (IAMRAT), College of Medicine, University of Ibadan, Ibadan 200132, Nigeria; iomorhason-bello@com.ui.edu.ng; 7 Khasto Consultants, Nairobi P.O. Box 18690-00100, Kenya; pkhayombe@khastoconsultants.org; 8 Ascendant & Company Ltd., 515 Freetown Highway, Sima Town, Western Area, Waterloo, Sierra Leone; olufela.adeyemi@asendantandcompny.com; 9 Apolowil Consultants, 113A Fife Avenue, Harare, Zimbabwe; ernest@apolowilconsultants.com; 10 Global Health Security Department, Infectious Diseases Institute, Makerere University College of Health Sciences, Kampala P.O. Box 22418, Uganda; ilumu@idi.co.ug; 11 Kwame Nkrumah University of Science and Technology (KNUST), Kumasi, Ghana; amuasi@kccr.de; 12 iRIS Research Consortium, 6 Ashur Suites, North Legon, Accra, Ghana; theo.acheampong@gmail.com; 13 Faculty of Health Sciences, 31 Bophelo Road, Gezina, University of Pretoria, Pretoria 0084, South Africa; td2581@cumc.columbia.edu

**Keywords:** COVID-19, vaccine hesitancy, sub-Saharan Africa

## Abstract

Rates of vaccination against COVID-19 remain lower in sub-Saharan Africa than in other low and middle-income regions. This is, in part, attributed to vaccine hesitancy, mainly due to misinformation about vaccine origin, efficacy and safety. From August to December 2021, we gathered the latest experiences and opinions on four vaccine hesitancy-related areas (policies, perceived risk religious beliefs, and misinformation) from 12 sub-Saharan African researchers, four of whom have published about COVID-19 vaccine hesitancy. The authors included two political and business experts, six public health specialists, five epidemiologists, and four biostatisticians from ten sub-Saharan African countries( Cameroon, Ghana, Kenya, Liberia, Nigeria, Sierra Leone, South Africa, Tanzania, Uganda, and Zimbabwe). The authors’ overarching opinions were that political influences, religious beliefs and low perceived risk exists in sub-Saharan Africa, and they collectively contribute to COVID-19 vaccine hesitancy. Communication strategies should target populations initially thought by policy makers to be at low risk, use multiple communication avenues and address major concerns in the population.

## 1. Introduction

Globally, there had been 522 million COVID-19 cases and six million COVID-19-related deaths by the third week of May 2022 [1]. The African region has reported over nine million cumulative COVID-19 cases and 172,308 deaths since the pandemic started. COVID-19 vaccines have proved to be an effective solution to preventing morbidity and mortality. These are complemented by other non-pharmaceutical interventions such as mask-wearing, social distancing, and hand washing.

Public health experts in the African region are increasingly concerned about the low vaccination uptake in sub-Saharan Africa, with eight in ten African countries unlikely to have attained the mid-2022 target of 70% vaccination rate [2]. This is despite the commendable efforts from global initiatives such as COVAX and the African Vaccine Acquisition Trust (AVAT) to attain equitable COVID-19 vaccine access. Such low levels of uptake are in part attributed to the high vaccine hesitancy, varying from 33% of the population in Mali [3], 50% of the people in Zimbabwe [4] and Ghana [5], and 85% reported in Cameroon [6].

Vaccine mistrust issues, vaccine safety, and the lack of reliable information are observed barriers to the uptake of COVID-19 vaccines [4,5,6]. The higher rate of observed COVID-19 vaccine hesitancy in some sub-Saharan African countries compared to high-income countries has been attributed to perceptions of low vaccine effectiveness, perceived low risk of contracting SARS CoV-2, misinformation and a fear of side effects [7].

The intent to vaccinate against COVID-19 was higher in some countries such as Ghana [5]. However, the context changed after the vaccine was introduced, due to mistrust of the vaccine-manufacturing companies or countries [5], doubts about vaccine efficacy [6,7], and fear of severe adverse effects following vaccination [7]. In sub-Saharan Africa, misinformation is widespread [4,6], hence driving hesitancy [6].

The purpose of this commentary is to highlight the barriers to COVID-19 vaccination and propose some solutions to accelerate the attainment of COVID-19 vaccination targets in the sub-Saharan African region. 

## 2. Methods 

Three authors (SK, EK, CK) from Tanzania drafted the commentary concept note and methods. They also requested, via email, expert opinions and experiences from nine other experts from nine sub-Saharan African countries, including Cameroon, Ghana, Kenya, Liberia, Nigeria, Sierra Leone South Africa, Uganda, and Zimbabwe. Four of the selected authors have published about COVID-19 vaccine hesitancy. The authors included two political and business experts, six public health specialists, five epidemiologists and four biostatisticians. Seven of the authors were affiliated with consultancies, two with research organisations, and seven with university faculties. All authors provided input on hesitancy-related thematic areas from their countries about: health policies related to COVID-19 vaccines, religious beliefs, perceived risks of COVID-19 infection, and influences of social media. Three authors (SK, EK, and CK) consolidated the inputs into a final commentary and synthesized the information according to theme and country into a final manuscript, which all authors reviewed. 

## 3. Results 

**Policies promoting vaccine hesitancy:** One of the strategies to increase equitable vaccine access for low-and middle-income countries has been the COVAX free vaccine program. Whereas 74% (34/46) of sub-Saharan countries, including Kenya, Uganda, and Zimbabwe, had started vaccinating their populations in January 2021, other countries, including Tanzania, had neither joined COVAX nor commenced vaccination against COVID-19 as of June 2021 [8]. The initial denial of COVID-19 existence and the “eradication of COVID-19” by divine powers and an antivaccine sentiments from key government officials delayed the COVID-19 vaccination program rollout in Tanzania [8]. As a result, after the vaccine was introduced in August 2021, dispelling the rumours became difficult despite government efforts, including hosting a national-level launch of the COVID-19 vaccination campaign. 

At the beginning of the vaccination programs, some countries did not wish to be accountable for the vaccine related adverse effects; they insisted the program was optional and voluntary. For example, in Uganda and Tanzania, the official communication from the relevant health ministries and directorates did not distinguish voluntary from necessary vaccination. Requiring written consent before vaccination exonerated the government from any blame for any adverse effects but increased fear and suspicion in the population. However, realizing the hesitancies of policymakers, affluent groups, for instance the rich and diplomats began the importation of vaccines ahead of the government (Kenya and Tanzania). This led to an assortment of vaccine brands in these countries, making the provision of the desirable vaccination schedule and regulatory oversight practically impossible. 

Initially, the vaccine was reserved for at-risk group such as the elderly, and provided at limited distribution points. However, these distribution points were few compared to the target population. Also, turnout at the designated health facilities remained low. As a result, governments lifted the limitations and more distribution points opened (Cameroon, Ghana, Kenya, Nigeria, Sierra Leone, Tanzania, Uganda and Zimbabwe). For instance, in Cameroon, Kenya and Tanzania all, hospitals at regional and district levels were providing the vaccine. In Uganda, as of November 2021, several private facilities and small health centres were permitted to vaccinate.

**Religious beliefs:** In some countries in sub-Saharan Africa, faith-based groups, which make up a significant section of the population, resist health care, including vaccines [9]. Religious leaders are trusted sources of information; however, as the COVID-19 pandemic entered its second wave, religious leaders became divided on the decision to vaccinate. Some religious leaders openly got vaccinated on national TV while others used religious gatherings to advance antivaccine campaigns (Ghana, Kenya, South Africa, Tanzania, and Zimbabwe). Meanwhile, despite some sects having beliefs against vaccination in Uganda, they did not publicly express antivaccine sentiments for fear of prosecution by the government. In Zimbabwe, apostolic sects have a history of resistance to vaccination programs and vaccine acceptance for most of these sects remains low [10]. However, when sect leaders realized the seriousness of the pandemic at the peak of the second wave, many churches decided to opened their doors only to those vaccinated against COVID-19 (Zimbabwe). 

**Perceived low risk and complacency:** In the early phase of the pandemic, some scholars believed that sub-Saharan Africans were less vulnerable to COVID-19, which might have contributed to poor vaccine uptake [6,11].

The targeted vaccination program caused the rest of the population to think that the vaccine was not for them, as the messages delivered prioritised at-risk groups such as health care workers, persons over 50 years, security officers, teachers and those with chronic ailments or comorbidities (Cameroon, Uganda, Tanzania, Ghana and Kenya). Those left out were considered by policy makers to be at low risk; hence many did not see the importance of vaccination (Cameroon, Nigeria, Sierra Leone and Uganda). Some countries even embraced herbal medicine and steam inhalations as both protective and curative for COVID-19. Hence, the populations did not think that taking the vaccine was important as the herbal medicine (Cameroon, Uganda, Sierra Leone and Tanzania). 

**Social media and misinformation:** Vaccine myths, misconceptions, and the spread of misinformation via social media platforms [12], led to the rapid growth of anti-COVID-19 vaccine campaigns. This was enabled by challenges in COVID-19 health communication, such as the lack of access to accurate information and protracted lockdowns that subjected people to unreliable social media channels as the only source of information. 

Several circulating myths, misconceptions and rumours regarding the origins of SARS-CoV-2 and the dangers of the vaccines in the population have circulated widely on various social media platforms (Cameroon, Ghana, South Africa and Zimbabwe) despite stricter media laws prohibiting the circulation of misinformation through social media (Cameroon, Sierra Leone, Tanzania). Predominantly, the misinformation was associated with misinterpretation of scientific information (Cameroon, Ghana, and Kenya). In specific cases, the misinformation was directed to certain brands of vaccines as being ineffective (Cameroon, Uganda).

## 4. Suggested Solutions for Vaccine Hesitancy

We highlight below some high-level interventions that could address COVID-19 Vaccine hesitancy and improve vaccine uptakes in sub-Saharan Africa.

1. Governments, policymakers and health workers at all levels should be conversant with the scientific basis of the COVID-19 interventions. They should be able to explicitly counter rumours and adequately explain the facts. This includes, for example, addressing concerns about why the development of COVID-19 vaccines was hastened and reassuring the public about the effectiveness and safety of the vaccine.
2. Countries should strive to resolve the mistrust of COVID-19 vaccines by advocating and lobbying for technology transfer to foster local vaccine production. South Africa is already producing some vaccines, and the government of Kenya has commissioned the production of COVID-19 vaccines through local research institutes.
3. Countries should initiate a context-tailored approach to COVID-19 vaccine awareness initiatives and integrate them in existing structures and programs, including involving religious leaders. Additionally, ethnographic research is required to identify multifaceted community engagement interventions which could include a cocktail of approaches to health communications appropriate for specific age groups within the population.
4. Public health experts in sub-Saharan Africa should counter misinformation, targeting younger people who are not only the majority but also the heaviest social media users.
5. Health workers should proactively guide the community on seeking credible information about the COVID-19 vaccines from trustworthy sources.
